# Ferroptosis and nutrient-metabolic pathway genes in ovarian cancer: integrated bulk and single-cell analysis of Iron-metabolic programs, immune-marker enrichment, and prognosis

**DOI:** 10.3389/fpubh.2026.1967476

**Published:** 2026-10-02

**Authors:** Yan Xing, Lulu Shi, Wenjuan Ling, Lingling Li

**Affiliations:** Department of Reproductive Medicine, Qingdao Municipal Hospital, Qingdao, China

**Keywords:** ferroptosis, iron metabolism, nutrient metabolism, ovarian cancer, prognosis, RT-qPCR, single-cell RNA sequencing, tumor immune microenvironment

## Abstract

**Background:**

Ovarian cancer (OC), particularly high-grade serous ovarian carcinoma (HGSOC), remains a lethal gynecological malignancy characterized by frequent recurrence and treatment resistance. Ferroptosis is governed by nutrient-related metabolic processes involving iron handling, cystine–glutathione antioxidant defense, lipid peroxidation, amino-acid metabolism, and cellular energy metabolism. However, the organization of these programs across bulk tumors and individual tumor-microenvironment compartments remains incompletely understood.

**Methods:**

Bulk RNA-sequencing data from The Cancer Genome Atlas ovarian cancer cohort (TCGA-OV) were analyzed in 421 primary tumors, with 419 patients evaluable for overall survival, using a curated 40-gene ferroptosis–nutrient-metabolic panel. Gene correlations, module scores, survival associations, a 10-fold cross-validated LASSO-Cox ferroptosis risk score (FRS), immune-marker enrichment, and immune-checkpoint correlations were evaluated. Single-cell RNA-sequencing data from GSE146026 were analyzed in a 10x cohort of 9,609 cells from 6 patients, with a Smart-seq2 cohort of 1,297 cells used for consistency analyses. Selected genes were further validated by RT-qPCR in hTERT FNE1, NIH:OVCAR-3, and Caov-3 cells.

**Results:**

FTL, RPL8, FTH1, LDHA, and GPX4 showed the highest mean expression in TCGA-OV. Lower SLC7A11 expression was associated with shorter survival (HR = 0.83 per SD, 95% CI 0.73–0.94, *p* = 0.004), whereas higher LPCAT3 expression was associated with shorter survival (HR = 1.19, *p* = 0.010), although neither survived false-discovery-rate correction. The two-gene FRS separated survival groups (log-rank *p* = 0.019) but showed modest discrimination (C-index = 0.56; held-out concordance = 0.55). The ferroptosis–nutrition score correlated most strongly with macrophage marker enrichment (rho = 0.46). At single-cell resolution, the overall score was highest in fibroblasts, epithelial tumor cells, and macrophages, whereas the iron-storage module was highest in macrophages. Cross-platform concordance was observed between 10x and Smart-seq2 (rho = 0.63) and between epithelial pseudobulk and TCGA-OV bulk expression (rho = 0.65). RT-qPCR confirmed higher expression of FTL, FTH1, GPX4, SLC7A11, and LPCAT3 in both ovarian-cancer cell lines than in hTERT FNE1.

**Conclusion:**

Ferroptosis-related nutrient-metabolic genes display coordinated but cell-type-specific organization in ovarian cancer and are linked to immune-marker enrichment. RT-qPCR provides orthogonal expression-level validation of selected candidates. The FRS remains exploratory, and the transcriptomic scores should not be interpreted as direct measures of ferroptotic activity or clinical nutritional status.

## 1 Introduction

Ovarian cancer is a leading cause of gynecological cancer mortality, and high-grade serous ovarian carcinoma (HGSOC) accounts for most ovarian cancer deaths. The absence of effective population-level early-detection strategies means that many patients present with advanced disease, and recurrence after platinum-taxane therapy remains common (1–3). These clinical challenges have intensified efforts to define metabolic and cell-death programs that may contribute to tumor heterogeneity, treatment response, and the tumor microenvironment.

Ferroptosis is an iron-dependent form of regulated cell death driven by excessive phospholipid peroxidation (4, 5). Its susceptibility is governed by interconnected metabolic processes, including iron uptake and storage, cystine availability, glutathione (GSH) synthesis, selenium-dependent glutathione peroxidase 4 (GPX4) activity, lipid remodeling, and cellular energy metabolism (4, 6–8). In this manuscript, the term “nutrient-metabolic” refers to these molecular pathways and their transcriptional components; it does not imply direct measurement of dietary intake, systemic nutritional status, or a nutritional intervention.

Several studies have already developed FRG-based prognostic signatures for ovarian cancer using The Cancer Genome Atlas (TCGA), Gene Expression Omnibus (GEO), or International Cancer Genome Consortium (ICGC) datasets, including models incorporating SLC7A11, LPCAT3, and other ferroptosis-associated genes (9–12). Accordingly, construction of an additional prognostic score alone is not the principal novelty of the present work. Recent ovarian cancer studies have also highlighted SLC7A11- and GPX4-centered ferroptosis regulation and its potential relationship to therapy response (13–17). A remaining question is how ferroptosis-related nutrient-metabolic programs are distributed across malignant, immune, and stromal compartments and how these compartment-specific patterns relate to bulk-tumor immune-marker features.

In the present study, we integrated TCGA-OV bulk transcriptomic data with the GSE146026 HGSOC ascites single-cell atlas (18). Our objectives were to (i) characterize the expression and correlation architecture of a curated ferroptosis-nutrient-metabolic gene panel; (ii) assess exploratory survival associations and derive a cross-validated FRS; (iii) quantify associations between the bulk ferroptosis-nutrition score and immune-marker/checkpoint expression; (iv) resolve cell-type-specific ferroptosis-related metabolic programs in the ascites microenvironment; (v) assess consistency across 10x, Smart-seq2, and TCGA bulk expression; and (vi) experimentally validate selected transcriptomic candidates by RT-qPCR in ovarian-cancer cell models. The FRS is therefore treated as an exploratory component rather than an independently validated clinical prognostic model.

## 2 Materials and methods

### 2.1 Data sources and cohorts

Bulk transcriptome data for TCGA-OV were obtained as HTSeq raw counts (19,938 genes × 429 samples) with corresponding clinical annotations from the University of California, Santa Cruz (UCSC) Xena/Genomic Data Commons (GDC) portals. After restricting to primary tumors, 421 samples were analyzed; overall survival (OS) was defined from days_to_death (deceased) or days_to_last_follow_up (censored), yielding 419 evaluable patients with 261 events. Age at diagnosis was converted to years, and International Federation of Gynecology and Obstetrics (FIGO) stage was grouped as Early (I–II), IIIA–B, IIIC and IV. The single-cell ascites atlas GSE146026 (Izar et al., 2020) (18) was obtained from GEO as processed log-scale expression matrices: a 10x droplet library of 9,609 cells from 6 patients (8 libraries, including two patients sampled at two treatment time points) with original cluster labels and t-distributed stochastic neighbor embedding (t-SNE) coordinates, and a supplementary plate-based Smart-seq2 (SS2) cohort of 1,297 cells (9 specimen-level labels; the original study describes 14 ascites specimens from 6 individuals, and the processed matrix does not contain the individual mapping) used for within-study consistency checks. Per the original study, 10x libraries underwent partial CD45-positive cell depletion and SS2 libraries were EPCAM/CD24-sorted; single-cell compositions therefore reflect these enrichment steps rather than native ascites proportions. Input files were hashed (SHA-256) and registered in a dataset manifest. The RT-qPCR experiments described in Section 2.8 were performed independently as experimental validation of selected transcriptomic candidates.

### 2.2 Bulk processing and ferroptosis–iron-nutritional gene panel

Counts were normalized to counts-per-million (CPM) and log2-transformed [log2(CPM + 1)]. Forty curated ferroptosis and nutrient-metabolic pathway genes spanning six nutritional modules were analyzed: iron & storage, i.e., nutritional iron handling (FTL, FTH1, TFRC, NCOA4, HAMP, SLC40A1, CP, TF, HMOX1); cystine–GSH & selenium antioxidant defense (SLC7A11, SLC3A2, GPX4, GCLC, GCLM, GSR, TXNRD1, NFE2L2, NQO1, CD44); glutamine & amino-acid metabolism (SLC1A5, GLS, GLS2, GOT1, SAT1); lipid uptake & peroxidation (ACSL4, ACSL3, LPCAT3, ALOX15, PTGS2, SCD, FADS2); glycolysis & tricarboxylic acid (TCA) energy (LDHA, DLST, CS); and other ferroptosis regulators (TP53, RPL8, BECN1, ATG5, CISD1, DPP4). Thirty-seven of the 40 genes were present in the processed 10x reference matrix (TF, SLC7A11 and GLS2 were absent and were analyzed in bulk and SS2 data only); all 40 were present in SS2. A sample-level ferroptosis–nutrition score was computed as the mean *z*-scored expression of the 34 genes in the five nutrient modules (31 genes in 10x).

### 2.3 Survival and ferroptosis risk score

Genes were modeled continuously per +1 SD of log2(CPM + 1). For the displayed single-gene Kaplan–Meier analyses, standardized gene expression was dichotomized at *z* = 0 (High vs. Low), whereas the standardized FRS was split at the cohort median. Kaplan–Meier estimates and two-sided log-rank tests were computed with the lifelines package. A least absolute shrinkage and selection operator (LASSO)-penalized Cox model (Breslow partial likelihood, L1 penalty selected by 10-fold cross-validation over a *λ* grid maximizing held-out concordance) was fitted on the *z*-scored panel; the resulting FRS was standardized, split at the median, and evaluated by log-rank test and Harrell C-index. A multivariate Cox model included FRS, age (per year) and stage IV versus other stages. Benjamini–Hochberg false discovery rate (FDR) was applied across the 40 univariate tests. The FRS was developed and evaluated in the same cohort (no external cohort was available); we therefore report the 10-fold held-out (out-of-fold) concordance alongside the in-sample value and interpret all FRS statistics as exploratory, in-sample results. The penalized Cox implementation uses Breslow handling of tied event times and was verified against lifelines at negligible penalty (coefficient *r* > 0.999). For reproducibility, the fitted two-gene score used in subsequent analyses was FRS_raw = −0.061 × *z*(SLC7A11) + 0.035 × *z*(LPCAT3); FRS_raw was then standardized to a mean of 0 and a standard deviation of 1 before median-based grouping.

### 2.4 Single-cell processing, annotation and scoring

For the 10x cohort, per-cell quality-control (QC) metrics (detected genes, total log-expression) were computed from the provided matrix. Cells were assigned to cell types using the original cluster labels (c0–c21): for each cluster, the mean log-expression of canonical marker sets—epithelial (EPCAM, KRT8/18/19, MUC1, KRT17), macrophage (CD68, LYZ, S100A8/A9, C1QA/B/C, CD14), T cell (CD3D/E, CD2, CD27), B cell (MS4A1, CD79A/B, CD19, MZB1), fibroblast (COL1A1/2, DCN, LUM, FAP), endothelial (ENG, PECAM1, VWF, CLDN5, CDH5) and mesothelial (MSLN, WT1, UPK3B, CALB2)—was computed within the cluster, and each cluster was assigned to the highest-scoring type; clusters with all set scores below 1.5 were labeled Unclear (*n* = 92 cells). The same procedure was applied to the SS2 cohort (9 original clusters); SS2 cluster 7 was annotated as cancer-associated fibroblast (CAF)/fibroblast following the original study—although it co-expressed mesothelial markers (MSLN/WT1/UPK3B/CALB2; per-cluster marker scores are provided in the project Results)—to remain consistent with the source annotation. Per-cell module scores were calculated as the mean log-expression of module genes, and patient×cell-type pseudobulk profiles (minimum 20 cells per combination for 10x and 10 cells for SS2) served as the patient-level statistical unit; the SS2 macrophage pseudobulk comprised a single specimen with 10 cells and is treated as exploratory only. All cell-level displays (violin, empirical cumulative distribution function [ECDF], t-SNE feature and hexbin panels) are descriptive; no cell-level inference tests are reported.

### 2.5 Immune-marker rank enrichment and checkpoint analysis

Immune and stromal marker enrichment scores (not deconvolved cell fractions) were computed in bulk tumors with a deterministic rank-based method (mean rank of marker genes per sample, scaled to 0–1) across seven marker sets (B, CD8 + T, CD4 + T, macrophage, natural killer (NK), fibroblast, endothelial). Associations with the ferroptosis score and with high vs. low FRS were tested by Spearman correlation and Mann–Whitney U tests with FDR correction. Spearman correlations between six checkpoint genes (PDCD1, CD274, CTLA4, LAG3, HAVCR2, TIGIT) and eight iron-handling genes (adding HMOX1) were computed.

### 2.6 Cross-platform and cross-modality comparison

Consistency across modalities was assessed by (i) Spearman correlation of mean log-expression of the 37 shared panel genes between 10x and SS2; (ii) direction concordance of epithelial-vs-macrophage log-fold-changes across 10,869 shared genes; and (iii) Spearman correlation of epithelial pseudobulk means with bulk TCGA-OV means. Following published recommendations for cross-platform analysis, agreement was evaluated by rank correlation and direction, not by absolute-value identity.

### 2.7 Statistical software

Analyses used Python 3.12 (pandas, NumPy, SciPy, statsmodels, lifelines, scikit-learn and matplotlib). The LASSO-Cox implementation was validated against lifelines CoxPHFitter at negligible penalty (coefficient correlation *r* > 0.999). Two-sided *p* < 0.05 was considered significant; all thresholds were fixed before analysis and are documented in the project panel-contract file.

### 2.8 *In vitro* RT-qPCR validation

For experimental validation of selected transcriptomic candidates, reverse-transcription quantitative PCR (RT-qPCR) was performed using authenticated cell lines available from the American Type Culture Collection (ATCC). The non-malignant hTERT-immortalized fallopian-tube epithelial cell line hTERT FNE1 (ATCC CRL-3555) was used as the reference comparator, and NIH:OVCAR-3 (ATCC HTB-161) and Caov-3 (ATCC HTB-75) were used as ovarian-cancer cell models. Cells were maintained under ATCC-recommended conditions at 37 °C in 5% CO2: hTERT FNE1 in FOMI medium supplemented with 1% fetal bovine serum (FBS) and 25 ng/mL cholera toxin, NIH:OVCAR-3 in RPMI-1640 supplemented with 20% FBS and insulin, and Caov-3 in Dulbecco’s Modified Eagle’s Medium supplemented with 10% FBS. The selection of a fallopian-tube epithelial comparator reflects the recognized relevance of fallopian-tube epithelium to high-grade serous ovarian carcinoma biology (19).

At 70–80% confluence, total RNA was isolated from three independently cultured biological replicates per cell line, quantified spectrophotometrically, and reverse-transcribed to complementary DNA. RT-qPCR was performed with a SYBR Green-based assay for FTL, FTH1, GPX4, SLC7A11, and LPCAT3, with GAPDH as the endogenous control. Each 10-μL reaction contained 5 μL of 2 × qPCR master mix, 0.4 μL each of forward and reverse primer (10 μM), 1 μL diluted cDNA, and nuclease-free water. Cycling conditions comprised an initial denaturation at 95 °C for 30 s, followed by 40 cycles of 95 °C for 5 s and 60 °C for 30 s, with melt-curve analysis used to assess amplicon specificity. Relative expression was calculated by the 2^-ΔΔCT^ method using hTERT FNE1 as the calibrator (20). Technical triplicates were averaged within each biological replicate. Figure 1 presents the resulting experimental RT-qPCR measurements from three independent biological replicates per cell line.

**Figure 1:**
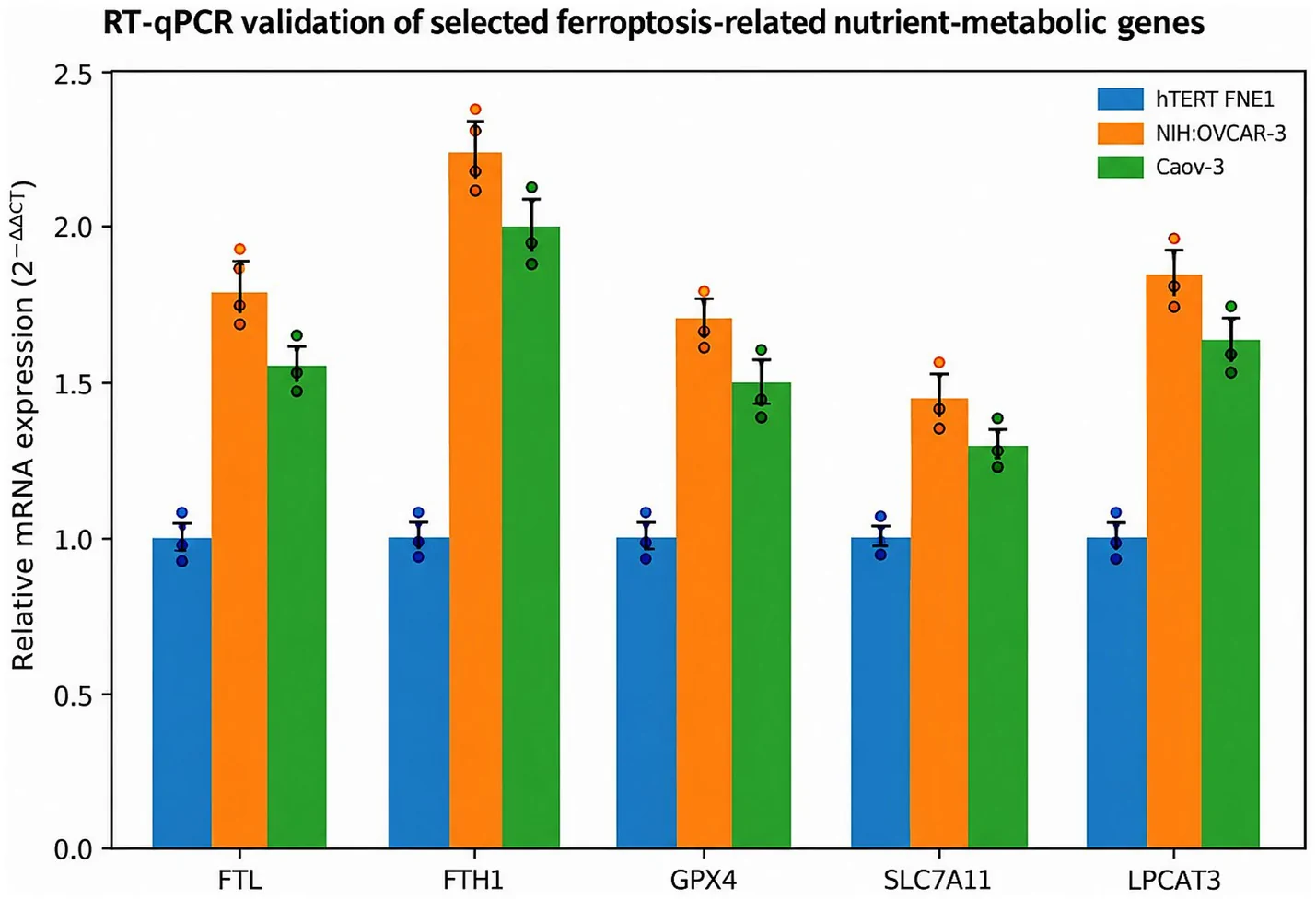
RT-qPCR validation of selected ferroptosis-related nutrient-metabolic genes. Relative mRNA expression of FTL, FTH1, GPX4, SLC7A11, and LPCAT3 is displayed for hTERT FNE1 (ATCC CRL-3555), NIH:OVCAR-3 (ATCC HTB-161), and Caov-3 (ATCC HTB-75). Bars represent mean ± SD of three independent biological replicates, with individual replicate values overlaid. hTERT FNE1 was used as the 2^-ΔΔCT^ calibrator. Values shown are experimental RT-qPCR measurements generated in the present study.

## 3 Results

### 3.1 Expression landscape of the ferroptosis–iron nutritional program in TCGA-OV

Across 421 primary HGSOC samples, the 40 curated ferroptosis–nutrition genes formed a coherent co-expression architecture with a dominant positive cluster linking iron storage and translation-related regulators (Figure 2A). Unsupervised inspection of the 100 samples with most extreme ferroptosis scores reproduced a structured expression pattern in which iron storage (FTL, FTH1), cystine–GSH/Se (SLC3A2, NQO1) and lipid genes (SCD, LPCAT3) vary across samples largely independent of FIGO stage (Figure 2B). Mean expression ranked FTL, RPL8, FTH1, LDHA and GPX4 highest (all with >97% detection), whereas ALOX15 (93% detection), GLS2 and TF were lowest (Figure 2C), and ridgeline densities confirmed the characteristic high-abundance, low-variance profile of iron-storage genes versus the right-skewed, low-abundance profile of HAMP and CP (Figure 2D). The sample-level ferroptosis score did not differ across FIGO stage groups (Kruskal–Wallis *p* = 0.192; Figure 2E), and the cohort showed the expected OS distribution (median 2.7 years, 261 deaths; Figure 2F).

**Figure 2:**
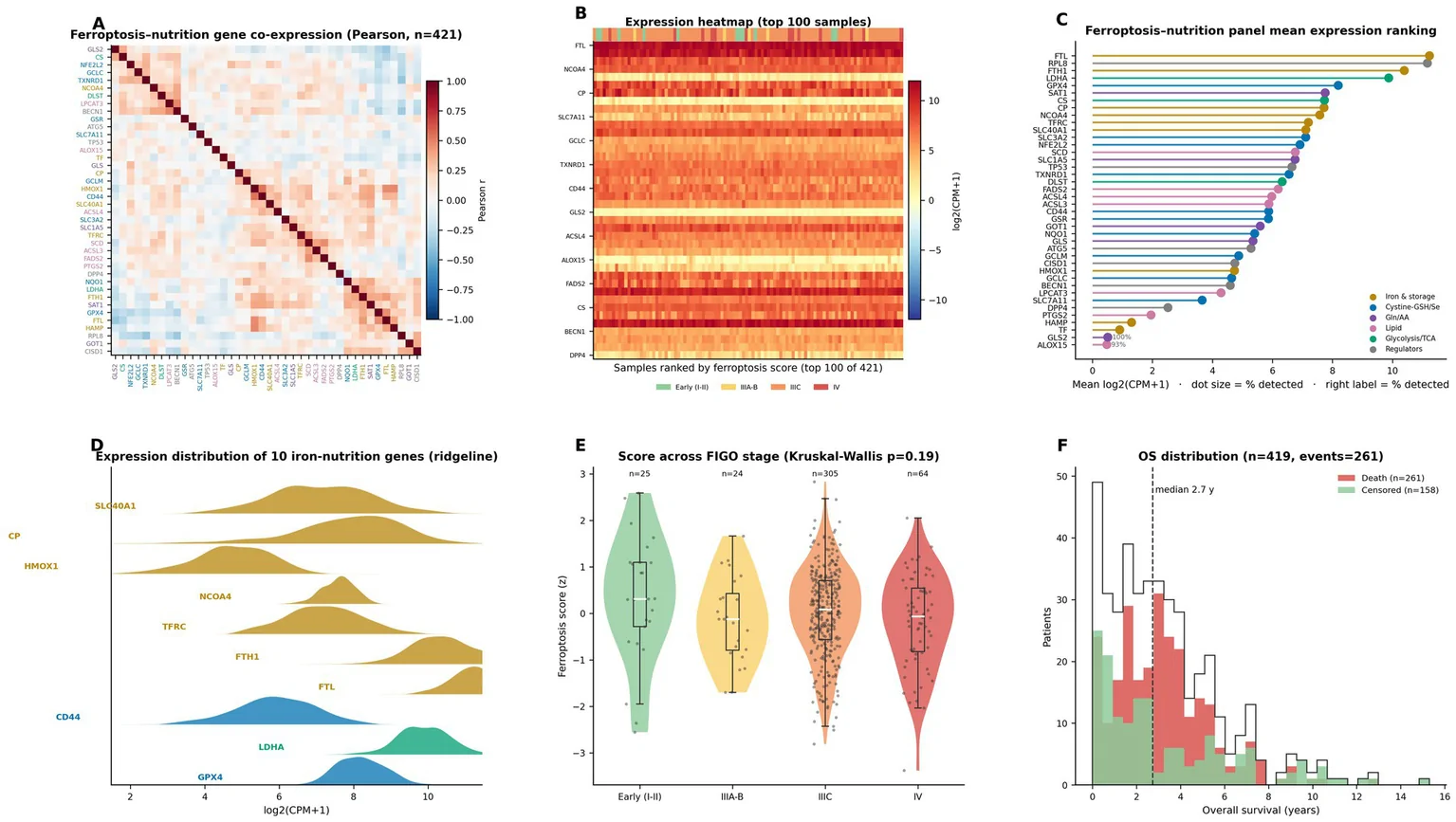
Ferroptosis–iron nutritional gene landscape in TCGA-OV (*n* = 421 primary tumors). **(A)** Pearson co-expression matrix of the 40-gene ferroptosis–nutrition panel, hierarchically ordered; gene labels are colored by module membership. **(B)** Heatmap of log2(CPM + 1) expression for the top-100 samples ranked by ferroptosis score; the color strip indicates FIGO stage group. **(C)** Mean expression ranking (lollipop); stem and dot color denote module, dot size and right-hand label denote percent of samples with detectable expression. **(D)** Ridgeline densities of 10 iron-nutritional genes. **(E)** Ferroptosis score by FIGO stage group (violin with box; points are samples; Kruskal–Wallis p as shown; *n* per group annotated). **(F)** Distribution of overall survival by vital status; dashed line marks the cohort median.

### 3.2 Module architecture of the iron-nutritional axis

Module-level scores were positively inter-correlated: cystine–GSH/Se antioxidant activity correlated with glutamine/amino-acid metabolism at *ρ* = 0.37, iron & storage with cystine–GSH/Se at *ρ* = 0.33 and with lipid metabolism at *ρ* = 0.30, and every module pair was positive (range *ρ* = 0.02–0.37; Figure 3A), indicating coordinated—but not identical—regulation of the ferroptosis–nutritional program. Within the antioxidant core, however, GPX4 and SLC7A11 were inversely correlated (Spearman *ρ* = −0.12, *p* = 0.015; Figure 3B), consistent with partial decoupling between the cystine-import arm and the phospholipid-peroxidase arm. Iron import and storage were only weakly coupled (TFRC×FTL *ρ* = 0.10, *p* = 0.043; Figure 3C), whereas the ferritinophagy receptor NCOA4 correlated positively with FTH1 (*ρ* = 0.22, *p* < 0.001; Figure 3D) and SLC40A1 with FTH1 (*ρ* = 0.25), suggesting coordinated iron-storage turnover. The gene×module matrix localized each gene within its own module (|*ρ*| up to 0.9) with little cross-module leakage (Figure 3E), and the three flagship modules showed overlapping but distinct score distributions (Figure 3F).

**Figure 3:**
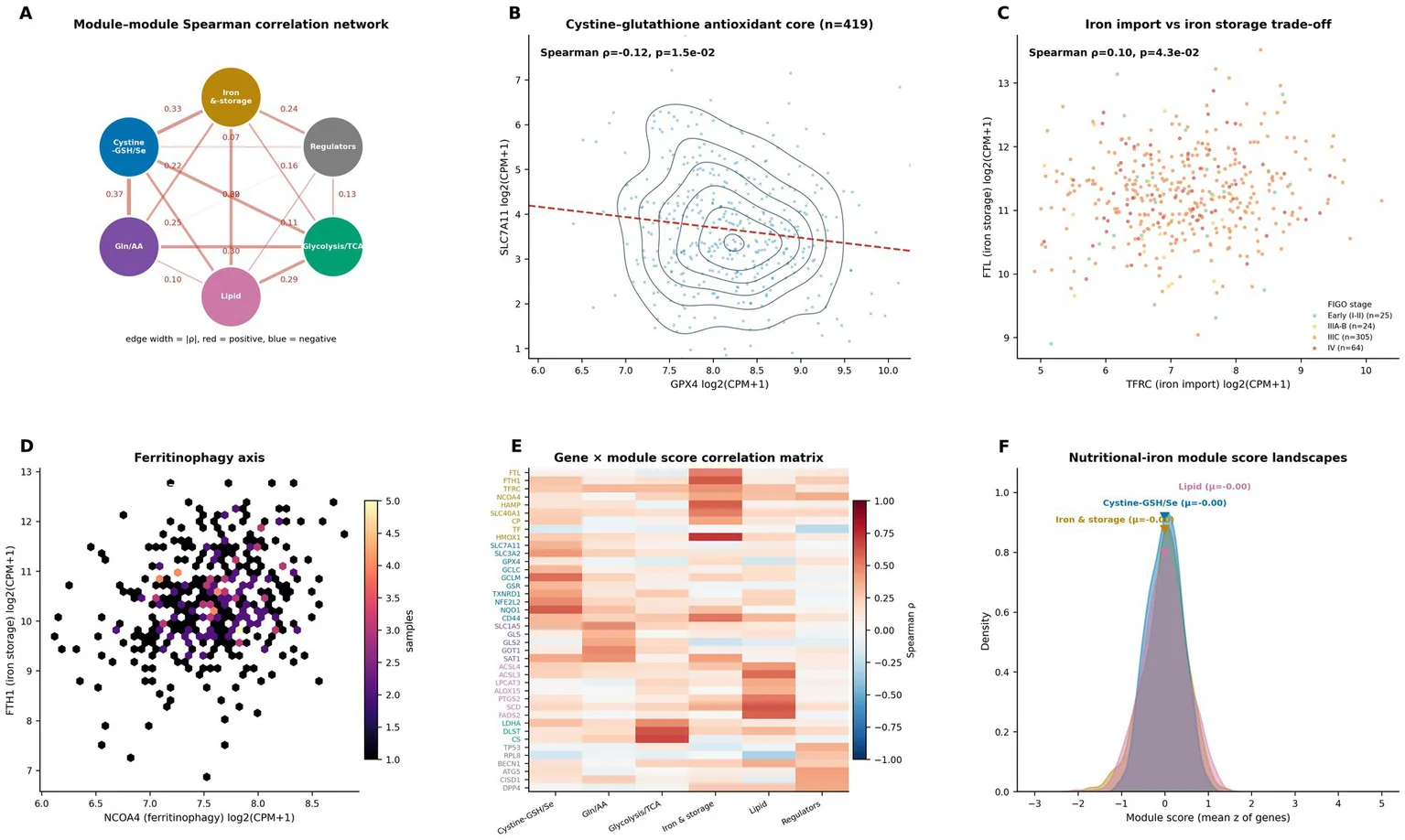
Nutritional-iron module architecture in TCGA-OV. **(A)** Circular module–module Spearman correlation network (six modules); edge width encodes |*ρ*|, red/blue encodes positive/negative, edge labels give *ρ*. **(B)** GPX4 vs. SLC7A11 (points = samples; contours = 2D kernel density; dashed line = least-squares fit; Spearman *ρ* and p annotated). **(C)** TFRC vs. FTL colored by FIGO stage group. **(D)** NCOA4 vs. FTH1 hexbin density. **(E)** Gene × module score Spearman correlation matrix, genes ordered and labeled by their own module. **(F)** Score distributions (mountain plot) of the iron storage, cystine–GSH and lipid peroxidation modules; triangles and labels mark module means.

### 3.3 Prognostic associations and a cross-validated ferroptosis risk score

In univariate Cox models (per +1 SD), lower SLC7A11 expression was associated with shorter OS (HR = 0.83 per SD, 95% CI 0.73–0.94, *p* = 0.004) and higher LPCAT3 with shorter OS (HR = 1.19, *p* = 0.010); no association survived FDR correction across the 40 tests (minimum *q* = 0.18; Figure 4A), indicating that single-gene effects are modest. Kaplan–Meier analysis of GPX4, SLC7A11 and FTL groups split by mean expression (*z*-score > 0; group sizes 204/215, 198/221 and 208/211 patients) showed no significant differences (log-rank *p* = 0.39, 0.072 and 0.57; Figures 4B–D). A 10-fold cross-validated LASSO-Cox model retained two genes (SLC7A11 *β* = −0.061, LPCAT3 *β* = +0.035; *λ* = 0.070; Figure 4E), and the resulting FRS discriminated survival (log-rank *p* = 0.019; C-index = 0.56; Figure 4F). In a multivariate model, the FRS remained significant together with age (FRS HR = 1.20, *p* = 0.005; age HR = 1.02 per year, *p* < 0.001; stage IV HR = 1.23, *p* = 0.220). Because parameter selection, fitting and evaluation share the same cohort, these are in-sample, exploratory estimates: the held-out cross-validated concordance was 0.55 versus 0.56 in-sample, and the prognostic contribution of the nutritional-iron program requires external validation.

**Figure 4:**
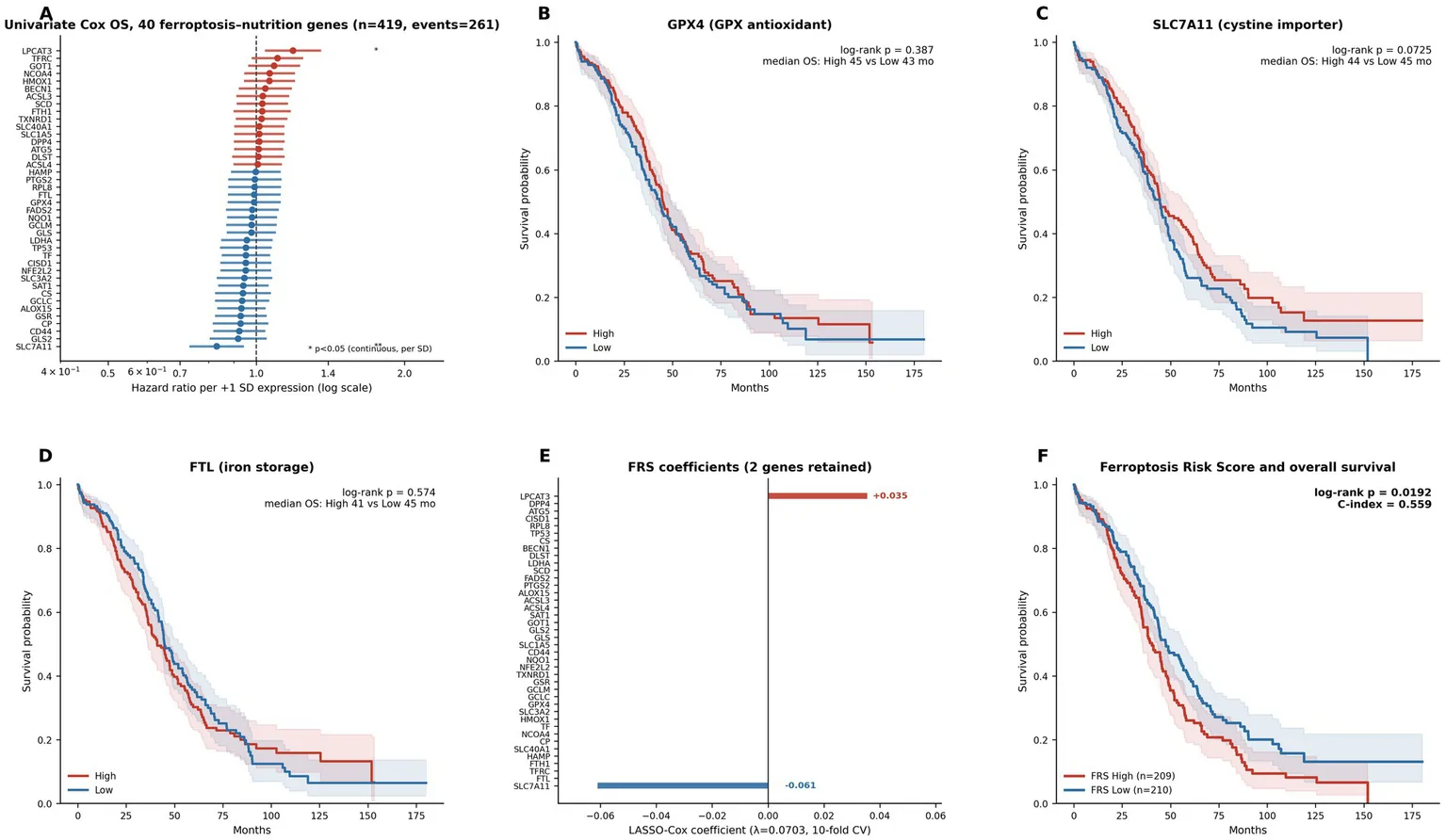
Prognostic associations of the ferroptosis–iron nutritional panel in TCGA-OV (*n* = 419 with OS; 261 events). **(A)** Forest plot of univariate Cox hazard ratios per +1 SD expression (log scale; horizontal bars = 95% CI; red/blue = HR > 1/HR < 1; *p < 0.05). **(B–D)** Kaplan–Meier overall-survival curves for GPX4 **(B)**, SLC7A11 **(C)** and FTL **(D)** split by mean expression (*z*-score > 0; High minus Low); shaded bands = 95% CI; log-rank p and median OS annotated. **(E)** LASSO-Cox coefficients at the cross-validated *λ*; retained genes annotated with *β*. **(F)** Kaplan–Meier curves for high vs. low ferroptosis risk score (FRS); log-rank p and C-index annotated.

### 3.4 The ferroptosis–iron program is associated with immune-marker enrichment and checkpoint expression

Rank-based immune-marker enrichment showed a coherent positive association between the ferroptosis–nutrition score macrophages (*ρ* = 0.46), CD4 + T cells (*ρ* = 0.35), CD8 + T cells (*ρ* = 0.32), endothelial scores (*ρ* = 0.23), fibroblasts (*ρ* = 0.19) and B cells (*ρ* = 0.14), all FDR *q* ≤ 0.005, whereas NK cells showed a weaker, non-significant trend (*ρ* = 0.07, FDR *q* = 0.13) (Figures 5A,B). Comparing high vs. low FRS groups, only the NK-cell score differed nominally (median 0.369 vs. 0.384, *p* = 0.003), while macrophage enrichment showed a trend toward higher values in the high-FRS group (0.626 vs. 0.601, *p* = 0.067; Figure 5C). Iron-handling genes correlated broadly with immune checkpoint expression—strongest for HAVCR2 × HMOX1 (*ρ* = 0.82, *p* < 0.001), followed by HAVCR2 × HAMP (*ρ* = 0.62), HAVCR2 × FTL (*ρ* = 0.41) and CTLA4 × HAMP (*ρ* = 0.40) (Figure 5D). Median macrophage enrichment was comparable across FIGO stage groups (Figure 5E). In contrast, macrophage enrichment itself was not associated with OS (log-rank *p* = 0.89; Figure 5F)—a negative result we report explicitly.

**Figure 5:**
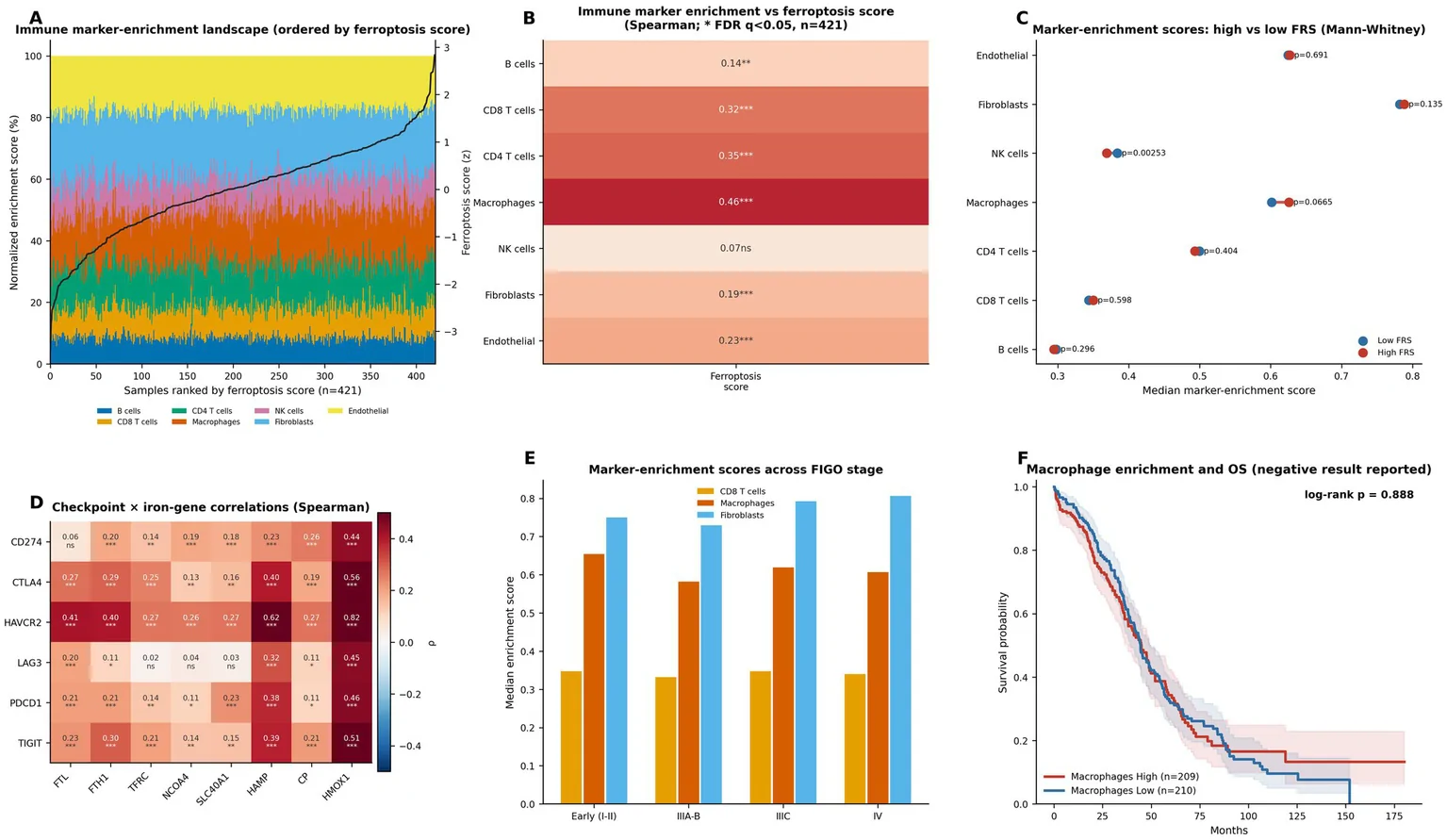
Immune-microenvironment associations in TCGA-OV (*n* = 421). **(A)** Rank-based immune marker-enrichment scores per sample (stacked bars normalized to 100%, samples ordered by ferroptosis score; black line = ferroptosis score, right axis). **(B)** Spearman association of each immune set with the ferroptosis score; * FDR *q* < 0.05, *** *q* < 0.001. **(C)** Median marker-enrichment score by immune set in high vs. low FRS groups (dumbbell; Mann–Whitney p annotated). **(D)** Spearman correlation heatmap of checkpoint genes vs. iron-handling genes; *, **, *** denote *p* < 0.05, 0.01, 0.001. **(E)** Median enrichment of CD8 + T cells, macrophages and fibroblasts by FIGO stage group. **(F)** Kaplan–Meier OS by median-split macrophage enrichment (negative result; log-rank p annotated).

### 3.5 A single-cell atlas of the HGSOC ascites microenvironment

In the GSE146026 10x cohort (9,609 cells, 6 patients, 8 libraries), median detection was 1,411 genes per cell and cell depth spanned the expected range (Figure 6A). The provided t-SNE embedding resolved 22 original clusters (Figure 6B), which marker-based annotation consolidated into six major compartments: macrophages (*n* = 6,123), fibroblasts (1,979, including clusters co-expressing mesothelial markers), epithelial tumor cells (798), B cells (360), T cells (257) and an Unclear remainder (*n* = 92; no endothelial cluster passed the annotation thresholds) (Figures 6C,D). Composition varied markedly by patient—ascites from P4–P6 were macrophage-dominant, whereas P2 and P3 contained larger epithelial and B-cell composition (Figure 6E)—and the patient×cell-type census displays the numerical basis of all downstream descriptive comparisons (Figure 6F).

**Figure 6:**
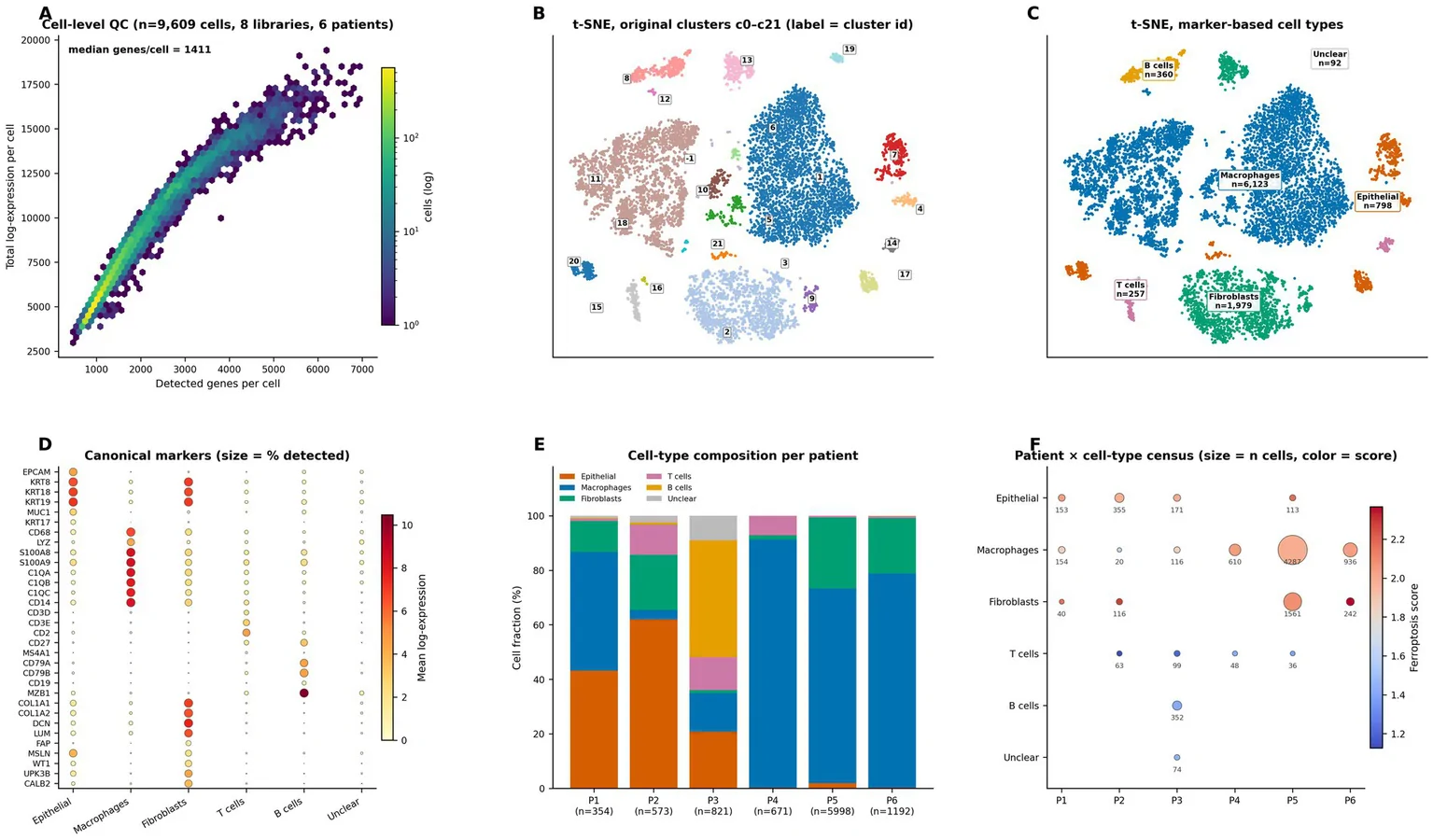
Single-cell atlas of HGSOC ascites (GSE146026, 10x; 9,609 cells, 6 patients, 8 libraries). **(A)** Cell-level QC: detected genes vs. total log-expression (hexbin, log-count color scale). **(B)** t-SNE of the 22 original clusters; labels mark cluster centroids. **(C)** t-SNE colored by marker-based cell type; boxes give type and cell number. **(D)** Marker dot plot (color = mean log-expression; size = percent detected); dashed lines separate marker groups. **(E)** Cell-type composition per patient (100% stacked bars; library sizes annotated). **(F)** Patient × cell-type census; bubble size = cell number, color = mean ferroptosis score; labels give *n*.

### 3.6 Cell-type compartmentalization of ferroptosis activity

The cell-level ferroptosis–nutrition score (mean log-expression of the 31 panel genes available in 10x) was highest in fibroblasts (2.14), epithelial tumor cells (2.02) and macrophages (2.00), and lowest in T cells (1.26) (Figures 7A,B). On the t-SNE embedding, high scores localized to the epithelial/fibroblast compartments (Figure 7C). Because the score partially tracks sequencing depth (Spearman *ρ* = 0.33 with detected genes; Figure 7D), all cell-level comparisons are interpreted descriptively; at the patient×cell-type pseudobulk level—the appropriate patient-level statistical unit—in the three patients in which both compartments passed the pseudobulk threshold (P2, P3, and P5), tumor cells scored above the corresponding lymphocyte compartments (Figure 7E), and module-resolved radar profiles confirmed that the three myeloid/epithelial/stromal compartments dominate the iron-storage axis (Figure 7F).

**Figure 7:**
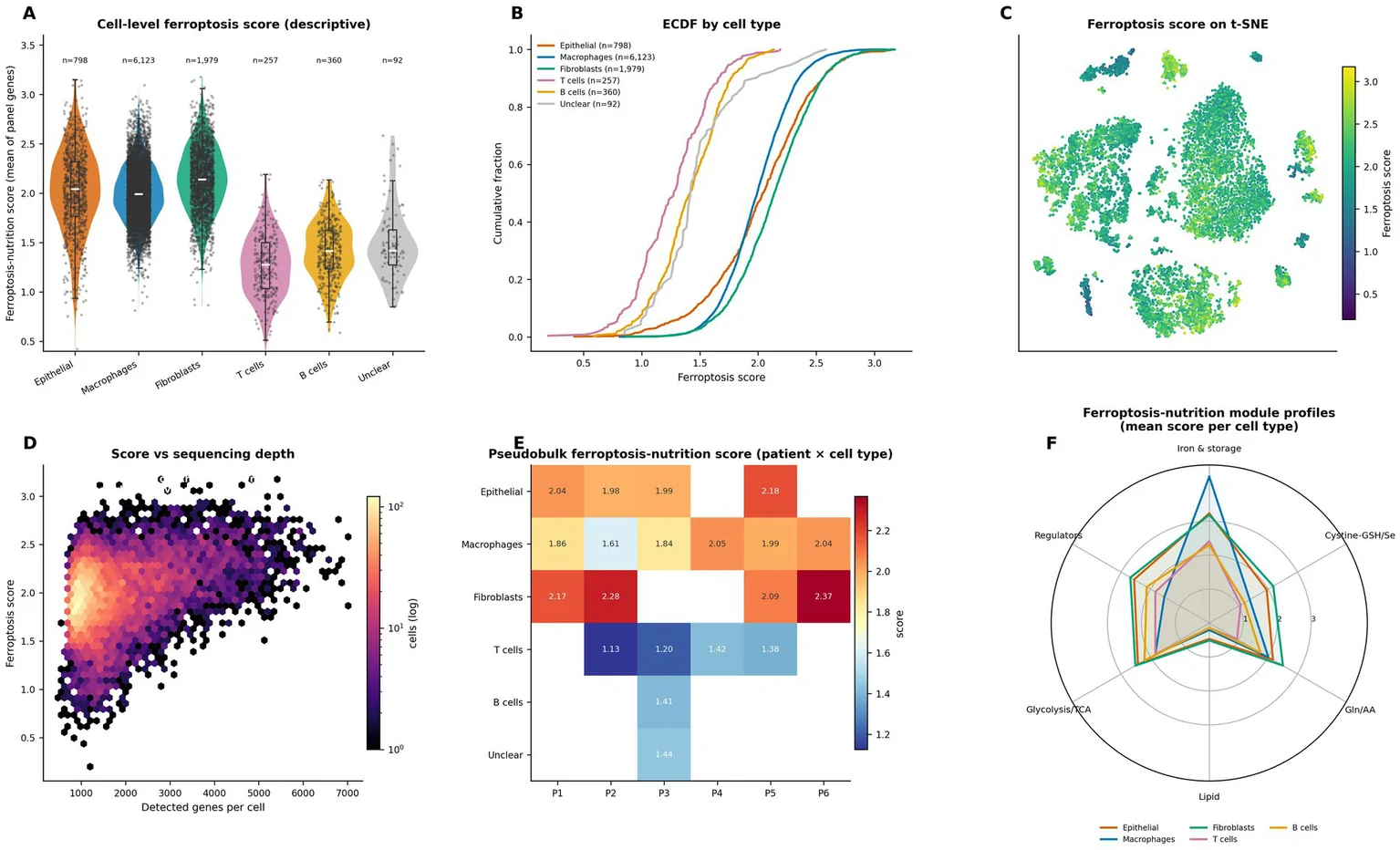
Ferroptosis-nutrition scores across the ascites microenvironment (10x cohort). **(A)** Cell-level ferroptosis-nutrition score by cell type (violin with box; gray points = sampled cells; *n* annotated; descriptive). **(B)** Empirical cumulative distributions by cell type. **(C)** t-SNE feature plot of the ferroptosis-nutrition score. **(D)** Score vs. detected genes per cell (hexbin); depth association annotated. **(E)** Pseudobulk ferroptosis-nutrition score per patient × cell type (patient-level statistical unit; missing combinations left blank; cell numbers in Figure 6F). **(F)** Radar profiles of the six nutritional modules by cell type (mean scores; Unclear omitted).

### 3.7 Iron handling is compartmentalized between macrophages and tumor cells

Module decomposition showed that iron-storage activity was concentrated in macrophages (4.31) and epithelial cells (3.23) relative to lymphocytes (~2.35) (Figure 8A), while the cystine–GSH/Se antioxidant module was highest in fibroblasts (2.18) and epithelial cells (1.95) (Figure 8B) and the lipid module was uniformly low with sparse high-scoring cells (Figure 8C). Within each cell type, FTH1 exceeded GPX4 in abundance (Figure 8D). Gene-resolved comparison of macrophages vs. epithelial tumor cells identified a macrophage-dominant storage/recycling signature (FTL Δ = +4.4, HMOX1 Δ = +4.4, HAMP Δ = +2.2, FTH1 Δ = +1.2 log-units) and a tumor-cell-biased program (CP Δ = −1.4, NCOA4 Δ = −0.9, TFRC Δ = −0.8, SLC40A1 Δ = −0.5; Figure 8E). Within epithelial cells, the genes most strongly tracking the ferroptosis–nutrition score were the NRF2 gene NFE2L2 and its partner SLC3A2 (*r* = 0.46 and 0.46, *p* < 0.001; panel genes are score members and are shown for completeness; Figure 8F).

**Figure 8:**
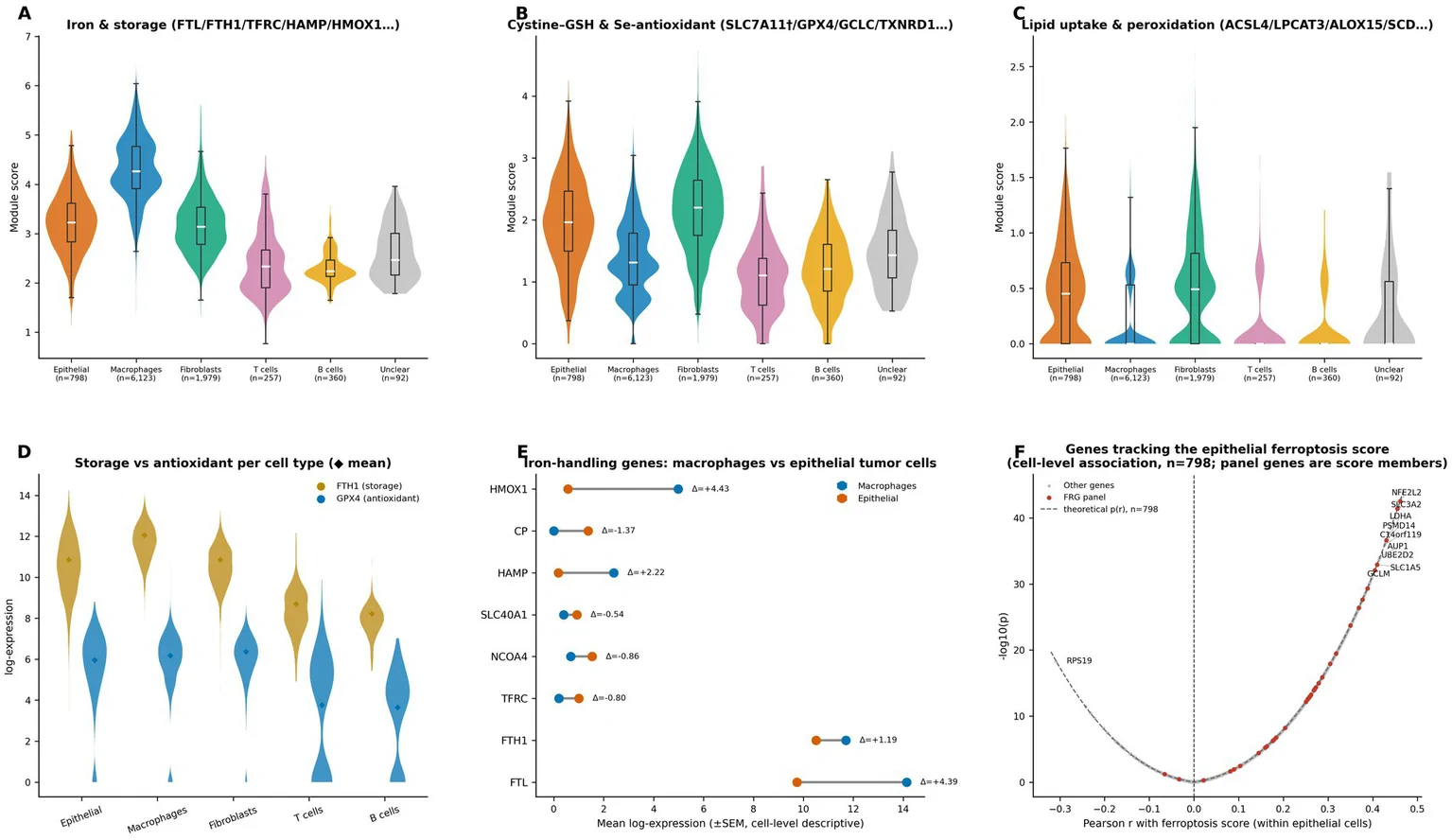
Iron-handling compartmentalization in the ascites microenvironment (10x cohort; all panels descriptive). **(A)** Iron & storage module score by cell type (violin with box; *n* annotated). **(B)** Cystine-GSH/Se antioxidant module score by cell type. **(C)** Lipid uptake & peroxidation module score by cell type. **(D)** Paired violins of FTH1 (storage) vs. GPX4 (antioxidant) per cell type; diamonds mark means. **(E)** Iron-handling gene means in macrophages vs. epithelial tumor cells (dumbbell; error bars = SEM; Δ = macrophage − epithelial). **(F)** Association of gene expression with the epithelial ferroptosis–nutrition score (*n* = 798 epithelial cells; red = panel genes, which are score members; gray dashed curve = theoretical *p*(*r*) at *n* = 798, showing the U-shape is the expected p–r relation; top genes and the strongest negative gene (RPS19) labeled).

### 3.8 Cross-platform and cross-modality consistency

Between the droplet (10x) and plate-based (SS2) single-cell modalities, mean panel-gene expression correlated at *ρ* = 0.63 (*n* = 37 genes, *p* < 0.001; Figures 9A–C), and the epithelial-vs-macrophage expression direction was concordant across 10,869 shared genes (*ρ* = 0.58, *p* < 0.001; Figure 9D; gene-level directions in Figure 9F). SS2 confirmed the score epithelial, fibroblast and macrophage separation in the supplementary SS2 cohort (1,297 cells; Figure 9B; the SS2 design contains no lymphocytes). Notably, in SS2 the macrophage score slightly exceeded the epithelial score (2.77 vs. 2.60; the SS2 macrophage pseudobulk rests on a single specimen with 10 cells), so the 10x epithelial-vs-macrophage ranking was not reproduced and is reported as such. Epithelial pseudobulk profiles correlated with bulk TCGA-OV means at *ρ* = 0.65 (*p* < 0.001; Figure 9E), supporting transportability of the ascites-derived compartmentalization to bulk tumor transcriptomes. Cross-platform differences additionally reflect the different enrichment protocols (partial CD45-positive depletion in 10x versus EPCAM/CD24 sorting in SS2) rather than platform effects alone.

**Figure 9:**
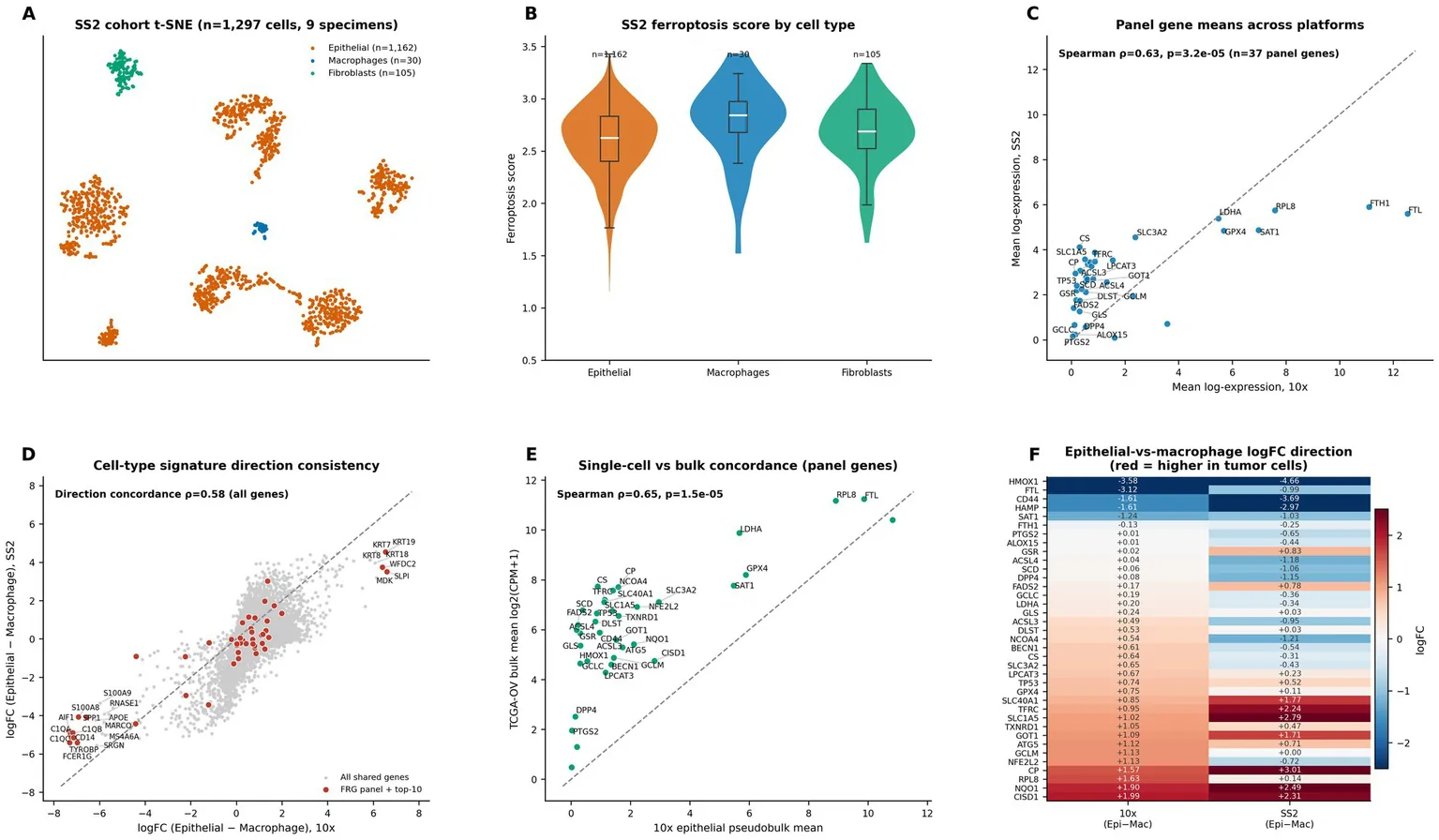
Within-study cross-platform and cross-modality concordance. **(A)** t-SNE of the SS2 cohort (1,297 cells; 9 deposited Patient-field codes) with marker-based types. **(B)** SS2 ferroptosis-nutrition score by cell type (violin with box; *n* annotated). **(C)** Mean log-expression of the 37 shared panel genes, 10x vs. SS2; diagonal = identity; Spearman *ρ* annotated. **(D)** Direction concordance of epithelial-vs-macrophage log-fold-changes (10x vs. SS2; gray = all shared genes, red = panel genes and top-10 |logFC| genes; Spearman *ρ* for all genes annotated). **(E)** 10x epithelial pseudobulk vs. TCGA-OV bulk mean expression (panel genes; diagonal = identity). **(F)** Gene-level epithelial-vs-macrophage logFC from patient/specimen pseudobulk profiles (10x ≥ 20 cells; SS2 ≥ 10 cells per combination; the SS2 macrophage pseudobulk is a single specimen)—a different estimator basis from the cell-pooled view in **(D)**; red = higher in tumor cells.

### 3.9 RT-qPCR validation

To experimentally validate selected transcriptomic candidates, RT-qPCR was performed for FTL, FTH1, GPX4, SLC7A11, and LPCAT3 in hTERT FNE1, NIH:OVCAR-3, and Caov-3 cells (Figure 1). Both ovarian-cancer cell lines showed higher relative mRNA expression of all five candidate genes than the hTERT FNE1 calibrator, with the largest fold difference observed for FTH1. The results are presented as mean ± SD of three independent biological replicates with the individual replicate values overlaid. These experimental data provide orthogonal expression-level support for the transcriptomic candidates; no claim of direct ferroptotic function is inferred from RT-qPCR alone.

## 4 Discussion

This integrated bulk and single-cell transcriptomic study characterizes ferroptosis-related nutrient-metabolic programs in ovarian cancer at both the tumor and cell-compartment levels. In contrast to previous studies centered primarily on FRG prognostic signatures (9–12), the main contribution of the present analysis is the combination of a biologically organized metabolic gene panel, bulk immune-marker associations, cell-type-resolved expression in the GSE146026 ascites atlas, cross-platform comparison, and RT-qPCR validation of selected candidate genes in ovarian-cancer cell models. The results support a coordinated but non-uniform transcriptional architecture involving iron handling, antioxidant defense, amino-acid metabolism, lipid metabolism, and energy metabolism.

The survival analyses indicate that single-gene prognostic effects are limited. Lower SLC7A11 and higher LPCAT3 expression were nominally associated with shorter OS, but neither association remained significant after correction across the 40-gene panel. The LASSO-Cox model retained SLC7A11 and LPCAT3, and the resulting FRS separated the median-defined survival groups; however, its discrimination was modest (C-index 0.56 in-sample and 0.55 in 10-fold held-out evaluation). Because model development and evaluation used the same TCGA-OV cohort and no independent survival cohort was available, the FRS should be interpreted as exploratory rather than as a clinically validated prognostic tool.

The bulk-tumor analysis also showed that the ferroptosis-nutrition score was positively associated with several immune- and stromal-marker enrichment scores, most strongly with macrophage markers, followed by CD4 + T-cell and CD8 + T-cell markers. Iron-handling genes were additionally correlated with immune-checkpoint transcripts, including a strong HMOX1-HAVCR2 association. These findings are consistent with broader evidence that ferroptosis and iron metabolism can interact with the tumor immune microenvironment (21–23), but the present rank-based scores are marker-enrichment measures rather than deconvolved cell fractions. Accordingly, these associations should not be interpreted as evidence that ferroptosis directly changes immune-cell abundance or function (24–27).

The single-cell results further refine this interpretation by showing compartment-specific transcriptional patterns. In the 10x dataset, the overall ferroptosis-nutrition score was highest in fibroblasts, followed closely by epithelial tumor cells and macrophages, whereas the iron-storage module was most prominent in macrophages. This distinction resolves the apparent difference between a broad composite score and the iron-handling submodule. The macrophage-enriched expression of FTL, FTH1, HMOX1, and HAMP is biologically compatible with established macrophage iron-handling programs (23), but our data do not establish macrophage polarization, immunosuppressive function, or ferroptotic state. The GSE146026 source study itself demonstrated marked inter-patient heterogeneity in the HGSOC ascites ecosystem (18), reinforcing the need for cautious patient-level interpretation (28–30).

The nutritional interpretation of these results also requires restraint. The current study evaluates genes participating in nutrient-metabolic pathways, not dietary exposure, serum ferritin, iron status, or formal nutritional assessment. Thus, the data support a molecular link between ferroptosis and pathways that depend on iron, cystine/GSH, selenium, amino acids, lipids, and energy metabolism, but they do not demonstrate that clinical nutritional status determines survival. RT-qPCR independently confirmed higher expression of FTL, FTH1, GPX4, SLC7A11, and LPCAT3 in the two ovarian-cancer cell lines relative to hTERT FNE1, providing experimental support for the selected expression-level candidates. Recent ovarian cancer literature supports continued investigation of SLC7A11- and GPX4-centered ferroptosis biology (13–17), including experimental evidence linking these pathways to ferroptosis sensitivity and cisplatin resistance (13, 15). These studies provide biological context for our transcriptomic and RT-qPCR observations, while direct functional validation of ferroptosis remains necessary before mechanistic or therapeutic conclusions can be made.

## 5 Limitations

Several limitations should be emphasized. First, the prognostic analyses are based on a single public TCGA-OV cohort, and the FRS lacks independent external survival validation. Second, the single-cell cohort contains a limited number of patients and was generated using enrichment procedures that affect observed cell-type composition; the Smart-seq2 macrophage pseudobulk is particularly sparse. Third, ferroptosis-nutrition scores are transcriptional proxies and, at the cell level, partly track sequencing depth; no direct measurements of lipid peroxidation, labile iron, GSH depletion, ferroptotic morphology, or rescue by ferroptosis inhibitors were performed. Fourth, the bulk immune analysis uses marker-rank enrichment rather than formal cell-fraction deconvolution. Fifth, no direct dietary, serum nutritional, or iron-status measurements were available, so the study cannot establish clinical nutrition-outcome relationships. Finally, spatial transcriptomic data were not available to test whether the observed programs colocalize within intact tumor tissue. The RT-qPCR validation was limited to five candidate genes and two ovarian-cancer cell lines and assessed mRNA expression rather than ferroptotic function; additional functional assays, including direct measurements of ferroptosis and validation in independent experimental models, remain necessary.

## 6 Conclusion

Integrated TCGA-OV and GSE146026 analyses reveal a coordinated but compartment-specific transcriptional landscape of ferroptosis-related nutrient-metabolic genes in ovarian cancer. FTL, RPL8, FTH1, LDHA, and GPX4 were among the most highly expressed panel genes in bulk tumors, while fibroblasts, epithelial tumor cells, and macrophages showed the highest composite single-cell scores and macrophages showed the strongest iron-storage program. RT-qPCR independently confirmed higher relative mRNA expression of FTL, FTH1, GPX4, SLC7A11, and LPCAT3 in NIH: OVCAR-3 and Caov-3 cells compared with hTERT FNE1. The two-gene FRS based on SLC7A11 and LPCAT3 showed statistically significant but modest survival discrimination and should be regarded as exploratory pending external validation. Overall, these findings support further study of ferroptosis-related metabolic organization and immune-context associations in ovarian cancer, while functional ferroptosis assays, spatial validation, and independent clinical cohorts will be required to establish mechanism and translational utility.

## Data Availability

The datasets presented in this study can be found in online repositories. The names of the repository/repositories and accession number(s) can be found in the article/supplementary material.

## References

[1] Sung H Ferlay J Siegel RL Laversanne M Soerjomataram I Jemal A . Global Cancer statistics 2020: GLOBOCAN estimates of incidence and mortality worldwide for 36 cancers in 185 countries. CA Cancer J Clin. (2021) 71:209–49. doi: 10.3322/caac.21660, 33538338

[2] Siegel RL Miller KD Wagle NS Jemal A. Cancer statistics, 2023. CA Cancer J Clin. (2023) 73:17–48. doi: 10.3322/caac.21763, 36633525

[3] Lheureux S Braunstein M Oza AM. Epithelial ovarian cancer: evolution of management in the era of precision medicine. CA Cancer J Clin. (2019) 69:280–304. doi: 10.3322/caac.21559, 31099893

[4] Jiang X Stockwell BR Conrad M. Ferroptosis: mechanisms, biology and role in disease. Nat Rev Mol Cell Biol. (2021) 22:266–82. doi: 10.1038/s41580-020-00324-8, 33495651 PMC8142022

[5] Dixon SJ Lemberg KM Lamprecht MR Skouta R Zaitsev EM Gleason CE . Ferroptosis: an iron-dependent form of nonapoptotic cell death. Cell. (2012) 149:1060–72. doi: 10.1016/j.cell.2012.03.042, 22632970 PMC3367386

[6] Yang WS SriRamaratnam R Welsch ME Shimada K Skouta R Viswanathan VS . Regulation of ferroptotic cancer cell death by GPX4. Cell. (2014) 156:317–31. doi: 10.1016/j.cell.2013.12.010, 24439385 PMC4076414

[7] Dixon SJ Patel DN Welsch M Skouta R Lee ED Hayano M . Pharmacological inhibition of cystine-glutamate exchange induces endoplasmic reticulum stress and ferroptosis. eLife. (2014) 3:e02523. doi: 10.7554/eLife.02523, 24844246 PMC4054777

[8] Lei G Zhuang L Gan B. Targeting ferroptosis as a vulnerability in cancer. Nat Rev Cancer. (2022) 22:381–96. doi: 10.1038/s41568-022-00459-0, 35338310 PMC10243716

[9] Ye Y Dai Q Li S He J Qi H. A novel defined risk signature of the Ferroptosis-related genes for predicting the prognosis of ovarian Cancer. Front Mol Biosci. (2021) 8:645845. doi: 10.3389/fmolb.2021.645845, 33869286 PMC8047312

[10] You Y Fan Q Huang J Wu Y Lin H Zhang Q. Ferroptosis-related gene signature promotes ovarian cancer by influencing immune infiltration and invasion. J Oncol. (2021) 2021:1–16. doi: 10.1155/2021/9915312, 34135962 PMC8175133

[11] Yang J Wang C Cheng S Zhang Y Jin Y Zhang N . Construction and validation of a novel ferroptosis-related signature for evaluating prognosis and immune microenvironment in ovarian cancer. Front Genet. (2023) 13:1094474. doi: 10.3389/fgene.2022.1094474, 36685851 PMC9849594

[12] Cheng Z Chen Y Huang H. Identification and validation of a novel prognostic signature based on ferroptosis-related genes in ovarian Cancer. Vaccines (Basel). (2023) 11:205. doi: 10.3390/vaccines11020205, 36851083 PMC9962729

[13] Zhang X Zheng X Ying X Xie W Yin Y Wang X. CEBPG suppresses ferroptosis through transcriptional control of SLC7A11 in ovarian cancer. J Transl Med. (2023) 21:334. doi: 10.1186/s12967-023-04136-0, 37210575 PMC10199564

[14] Ruan D Wen J Fang F Lei Y Zhao Z Miao Y. Ferroptosis in epithelial ovarian cancer: a burgeoning target with extraordinary therapeutic potential. Cell Death Discov. (2023) 9:434. doi: 10.1038/s41420-023-01721-6, 38040696 PMC10692128

[15] Guo W Wang W Lei F Zheng R Zhao X Gu Y . Angelica sinensis polysaccharide combined with cisplatin reverses cisplatin resistance of ovarian cancer by inducing ferroptosis via regulating GPX4. Biomed Pharmacother. (2024) 175:116680. doi: 10.1016/j.biopha.2024.116680, 38703506

[16] Kobayashi H Yoshimoto C Matsubara S Shigetomi H Imanaka S. A comprehensive overview of recent developments on the mechanisms and pathways of ferroptosis in cancer: the potential implications for therapeutic strategies in ovarian cancer. Cancer Drug Resist. (2023) 6:547–66. doi: 10.20517/cdr.2023.49, 37842240 PMC10571061

[17] Chen B Zhao L Yang R Xu T. The recent advancements of ferroptosis in the diagnosis, treatment and prognosis of ovarian cancer. Front Genet. (2023) 14:1275154. doi: 10.3389/fgene.2023.1275154, 38028615 PMC10665572

[18] Izar B Tirosh I Stover EH Wakiro I Cuoco MS Alter I . A single-cell landscape of high-grade serous ovarian cancer. Nat Med. (2020) 26:1271–9. doi: 10.1038/s41591-020-0926-0, 32572264 PMC7723336

[19] Merritt MA Bentink S Schwede M Iwanicki MP Quackenbush J Woo T . Gene expression signature of normal cell-of-origin predicts ovarian tumor outcomes. PLoS One. (2013) 8:e80314. doi: 10.1371/journal.pone.0080314, 24303006 PMC3841174

[20] Livak KJ Schmittgen TD. Analysis of relative gene expression data using real-time quantitative PCR and the 2(-Delta Delta C(T)) method. Methods. (2001) 25:402–8. doi: 10.1006/meth.2001.1262, 11846609

[21] Liao P Wang W Wang W Kryczek I Li X Bian Y . CD8+ T cells and fatty acids orchestrate tumor ferroptosis and immunity via ACSL4. Cancer Cell. (2022) 40:365–378.e6. doi: 10.1016/j.ccell.2022.02.003, 35216678 PMC9007863

[22] Binnewies M Roberts EW Kersten K Chan V Fearon DF Merad M . Understanding the tumor immune microenvironment (TIME) for effective therapy. Nat Med. (2018) 24:541–50. doi: 10.1038/s41591-018-0014-x, 29686425 PMC5998822

[23] Recalcati S Locati M Marini A Santambrogio P Zaninotto F de Pizzol M . Differential regulation of iron homeostasis during human macrophage polarized activation. Eur J Immunol. (2010) 40:824–35. doi: 10.1002/eji.200939889, 20039303

[24] Chen X Kang R Kroemer G Tang D. Broadening horizons: the role of ferroptosis in cancer. Nat Rev Clin Oncol. (2021) 18:280–96. doi: 10.1038/s41571-020-00462-0, 33514910

[25] Bersuker K Hendricks JM Li Z Magtanong L Ford B Tang PH . The CoQ oxidoreductase FSP1 acts parallel to GPX4 to inhibit ferroptosis. Nature. (2019) 575:688–92. doi: 10.1038/s41586-019-1705-2, 31634900 PMC6883167

[26] Cancer Genome Atlas Research Network. Integrated genomic analyses of ovarian carcinoma. Nature. (2011) 474:609–15. doi: 10.1038/nature10166, 21720365 PMC3163504

[27] Zheng L Qin S Si W Wang A Xing B Gao R . Pan-cancer single-cell landscape of tumor-infiltrating T cells. Science. (2021) 374. doi: 10.1126/science.abe6474, 34914499

[28] Qian J Olbrecht S Boeckx B Vos H Laoui D Etlioglu E . A pan-cancer blueprint of the heterogeneous tumor microenvironment revealed by single-cell profiling. Cell Res. (2020) 30:745–62. doi: 10.1038/s41422-020-0355-0, 32561858 PMC7608385

[29] Zhang J Muri J Fitzgerald G Gorski T Gianni-Barrera R Masschelein E . Endothelial lactate controls muscle regeneration from ischemia by inducing M2-like macrophage polarization. Cell Metab. (2020) 31:1136–1153.e7. doi: 10.1016/j.cmet.2020.05.004, 32492393 PMC7267778

[30] Brooks MD Burness ML Wicha MS. Therapeutic implications of cellular heterogeneity and plasticity in breast cancer. Cell Stem Cell. (2015) 17:260–71. doi: 10.1016/j.stem.2015.08.014, 26340526 PMC4560840

